# Role of adipose tissue-derived cytokines in the progression of inflammatory breast cancer in patients with obesity

**DOI:** 10.1186/s12944-022-01678-y

**Published:** 2022-08-04

**Authors:** Aya Saber Ibrahim, Mohamed El-Shinawi, Salwa Sabet, Sherif Abdelaziz Ibrahim, Mona Mostafa Mohamed

**Affiliations:** 1 Zoology Department, Faculty of Science, Cairo University, Giza, 12613 Egypt; 2 Department of General Surgery, Faculty of Medicine, Ain Shams University, Cairo, 11566 Egypt; 3 International Affairs, Galala University, Suez, Egypt; 4 Molecular Biotechnology Program, Faculty of Science, Galala University, Suez, Egypt

**Keywords:** Inflammatory breast cancer, Cancer-associated adipose tissue, Obesity, Interleukin-6, Interleukin-8, Monocyte chemo-attractant protein-1, Breast cancer stem cells, EMT

## Abstract

**Background:**

Inflammatory breast cancer (IBC) represents a deadly aggressive phenotype of breast cancer (BC) with a unique clinicopathological presentation and low survival rate. In fact, obesity represents an important risk factor for BC. Although several studies have identified different cellular-derived and molecular factors involved in IBC progression, the role of adipocytes remains unclear. Cancer-associated adipose tissue (CAAT) expresses a variety of adipokines, which contribute to tumorigenesis and the regulation of cancer stem cell (CSC). This research investigated the potential effect of the secretome of CAAT explants from patients with BC on the progression and metastasis of the disease.

**Methods:**

This study established an *ex-vivo* culture of CAAT excised from IBC (n = 13) *vs.* non-IBC (*n* = 31) patients with obesity and profiled their secretome using a cytokine antibody array. Furthermore, the quantitative PCR (qPCR) methodology was used to validate the levels of predominant cytokines at the transcript level after culture in a medium conditioned by CAAT. Moreover, the impact of the CAAT secretome on the expression of epithelial-mesenchymal transition (EMT) and cells with stem cell (CSC) markers was studied in the non-IBC MDA-MB-231 and the IBC SUM-149 cell lines. The statistical differences between variables were evaluated using the chi-squared test and unpaired a Student’s *t*-test.

**Results:**

The results of cytokine array profiling revealed an overall significantly higher level of a panel of 28 cytokines secreted by the CAAT *ex-vivo* culture from IBC patients with obesity compared to those with non-IBC. Of note, interleukin-6 (IL-6), interleukin-8 (IL-8), and monocyte chemo-attractant protein 1 (MCP-1) were the major adipokines secreted by the CAAT IBC patients with obesity. Moreover, the qPCR results indicated a significant upregulation of the *IL-6*, *IL-8*, and *MCP-1* mRNAs in CAAT *ex-vivo* culture of patients with IBC *vs.* those with non-IBC. Intriguingly, a qPCR data analysis showed that the CAAT secretome secretions from patients with non-IBC downregulated the mRNA levels of the *CD24* CSC marker and of the epithelial marker E-cadherin in the non-IBC cell line. By contrast, E-cadherin was upregulated in the SUM-149 cell.

**Conclusions:**

This study identified the overexpression of *IL-6*, *IL-8*, and *MCP-1* as prognostic markers of CAAT from patients with IBC but not from those with non-IBC ; moreover, their upregulation might be associated with IBC aggressiveness via the regulation of CSC and EMT markers. This study proposed that targeting IL-6, IL-8, and MCP-1 may represent a therapeutic option that should be considered in the treatment of patients with IBC.

**Supplementary Information:**

The online version contains supplementary material available at 10.1186/s12944-022-01678-y.

## Background

Breast cancer (BC) is the second leading cause of death among women globally. An estimated 1.7 million women have been diagnosed with BC, accounting for over 521,900 deaths worldwide [1, 2]. The number of BC-related death is expected to increase by 20% in the coming years, with over 95% of new cases being diagnosed over the age of 40 [3]. In Egypt, patients with BC present at a young age and have more aggressive properties than do patients in Western countries [4, 5].

Inflammatory breast cancer (IBC) is the most lethal subtype of BC [6–8]. IBC is diagnosed at a younger age compared with other types of BC [9]. Clinically, IBC is defined by distinct and painful clinical symptoms. For example, Patients with IBC have inflamed red or swollen breasts and a “peau d’orange” appearance [10–12]. However, IBC is still misdiagnosed as an infection [13].

Obesity is one of the strongest determinants of BC risk and survival rate [14]. Obesity is a condition in which the body’s fat levels are abnormally high and is defined as a body mass index (BMI) ≥ 30 kg/m2 [15, 16]. Obese patients with BC have a bigger tumor mass and a higher risk of lymphatic invasion as a consequence of obesity [17]; moreover, they have a poor prognosis and decreased overall survival compared with their lean counterparts [18–20]. Obesity promotes resistance to anticancer therapy in BC [21] by preventing the exposure of cancer cells to drugs, as reported previously [22, 23]. Moreover, the poor recurrence of BC may be adversely affected by obesity [24–26]. Because BC grows in the vicinity of adipose tissue (AT), AT dysfunction resulting from obesity is considered a critical determinant of cancer progression [27]. AT is a highly complex tissue that accounts for approximately 10% of the breast size [28, 29].

AT contains a heterogeneous population of cells, such as adipocytes, peri-adipocytes, adipose stromal/stem cells, fibroblasts, and endothelial cells [30–32]. As a result of excess calory intake, adipocytes begin to expand (hypertrophy), which in turn can induce an increase in adipose cell number (hyperplasia), to accommodate the excess energy [31], thus leading to obesity.

Typically, the principal function of AT is the storage of excess energy in the form of triglycerides [33, 34]. Moreover, it is a critical endocrine organ that expresses a multiplicity of determined biologically active molecules that control many systemic physiological processes [35, 36]. Collectively, adipokines or adipocytokines are the secretions of AT [36–38].

In BC, the alteration of the adipokine profile, including molecules such as *IL-6, IL-8, and MCP-1,* has a significant impact on cancer progression [2] as it promotes cancer proliferation and invasion, controls epithelial derived-proteins and the production of growth factors, and induces other cells of the tumor microenvironment (TME), to trigger invasion [35] via different mechanisms such as the induction of cancer stem cell-like traits and the modulation of the influencing epithelial–mesenchymal transition (EMT) [39]. The EMT, in which cancer cells lose their epithelial characteristics and acquire mesenchymal-like characteristics, is considered one to be of the essential steps in BC metastasis because it induces the acquisition of cells with stem cell (CSC) characteristics, which that can initiate new tumors [40].

In the present study, an *ex-vivo* culture of cancer-associated adipose tissue (CAAT) obtained from human patients with non-IBC and IBC during curative surgery was established, to mimic the *in-vivo* conditions of the TME. Moreover, to determine the predominant adipokines in this context, the mediators/adipokines secreted by the CAAT *ex-vivo* culture were profiled. Furthermore, their effect on the expression of CSC- and EMT-related genes in BC cell lines was studied.

## Methods

### Patient samples

For the purpose of patient enrolment in this study, Institutional Review Board (IRB#00006379) approval was obtained before surgical treatment. The experimental protocol was approved by the research ethics committee of the General Surgery Department, Faculty of Medicine, Ain Shams University, Cairo, Egypt. Patients who were preoperatively diagnosed as BC cases in the breast clinic of Ain Shams University Hospitals by clinical examination, mammography, ultrasound, and Tru-cut biopsy and declared their informed consent in written form prior to participation, including agreements for enrolment in this study and publication of data were enrolled in the study. Mammary AT was collected from 44 Egyptian BC patients with BMI ≥ 30 who underwent modified radical mastectomy or conservative surgery. The patients enrolled in this study were divided into two groups; the non-IBC (n = 31) and IBC (n = 13) groups who were selected for MRM surgical treatment. The clinical and pathological in the non-IBC and IBC groups who participated in the present study are listed in Table 1.

**Table 1:** Clinical and pathological data of obese breast cancer patients

Characteristic	Non-IBC (*N* = 31)	IBC (*N* = 13)	*P* value
Age (years)			
Range	36-71	30-69	0.656ª
Mean ± SEM	48.5 ± 1.5	46.23 ± 4.68	
Tumour size (cm)			
≤ 4	14 (45.2%)	6 (60%)	1^b^
> 4	8 (25.8%)	4 (40%)	
NA	9	3	
Lymph node status, n (%)			
≤ 4	19 (61.3%)	6 (50%)	0.29^b^
> 4	8 (25.8%)	6 (50%)	
NA	4	1	
Tumour grade, n (%)			
G1	0	0	0.65^b^
G2	23 (74.2%)	12 (92.3%)	
G3	4 (12.9%)	1 (7.7%)	
NA	4	0	
Lymphovascular invasion, n (%)			
Negative	10 (32.3%)	4 (40%)	0.7^b^
Positive	9 (29%)	6 (60%)	
NA	12	3	
ER, n (%)			
Negative	4 (12.9%)	2 (15.38%)	1^b^
Positive	26 (83.9%)	11 (84.62%)	
NA	1	0	
PR, n (%)			
Negative	5 (16.1%)	3 (23.08%)	0.68^b^
Positive	25 (80.6%)	10 (76.92%)	
NA	1	0	
HER-2, n (%)			
Negative	23 (74.2%)	8 (61.54%)	1^b^
Positive	7 (22.6%)	5 (38.46%)	
NA	1	0	

Data are expressed as mean ± SEM

*NA* not available

^*^significant *P*-value calculated by ªStudent’s t-test or ^b^Pearson Chi-Square

### Sample collection and handling

AT was obtained from the safety margin, i.e., 3–5 cm away from the resected mass, and no cancer cells were detected by the pathologist. All tissue specimens were collected in a medium and immediately transported in an ice box from the operation room to the laboratory within 1 h, for further experiments.

### Culture of cancer-associated adipose tissue (CAAT) and preparation of conditioned medium (CM)

The procedures for handling the tissue specimens were performed under aseptic conditions in the biological safety cabinet level II. CAAT (approximately 300 mg) specimens isolated from patients with non-IBC and IBC were washed with phosphate-buffered saline (PBS) twice and transferred to a 35 mm tissue culture dish containing a warm DMEM/F-12 medium supplemented with 10% fetal bovine serum (FBS) (Gibco, Waltham, Massachusetts, USA). Subsequently, the CAAT specimens were carefully minced with a sterile sharp scalpel into tiny pieces (approximately 0.5 mm in diameter) in a sterile 60 mm petri dish. The CAAT fragments were distributed equally, and a pre-warmed DMEM-F12 medium supplemented with 10% FBS was added and the dish was incubated for 24 h in a humidified atmosphere of 5% CO2 at 37°C. The following day, the medium was discarded, followed by starvation by adding 1 mL of serum-free culture medium and incubation for 24 h. Next, the CAAT-CM was collected and centrifuged at 800 *xg* at 4°C for 5 min, to separate it into the cell pellet and supernatant. The supernatant was collected and centrifuged at 2000 *xg* at 4°C for 10 min to eliminate cell debris, aliquoted, and stored at -80°C for cytokine profiling and conditioning breast cancer cell line culture medium. CAAT *ex-vivo* cultures were homogenized in TRI Reagent® (Sigma life science, Missouri, USA) to prepare the total RNA for real-time PCR experiments. Unless otherwise stated, all tissue culture medium were from Lonza (Basel, Switzerland), and supplies were from Greiner bio-one (Frickenhausen, Germany).

### Profiling of non-IBC and IBC CAAT secretomes using a human cytokine array

The Ray-Bio^TM^ cytokine antibody array-C3 (RayBiotech, Inc. GA, USA) was used to detect 42 different cytokines and adipokines in the CAAT-CM of patients in non-IBC and IBC groups. Briefly, the secretome of CAAT *ex-vivo* culture was concentrated 10-fold using Amicon Ultracell 10K filters (Millipore, Billerica, MA). The kit components were allowed to equilibrate at room temperature (RT). As described in the kit manual, the cytokine array membrane was incubated with 2 mL of blocking buffer for 1 h at RT. Subsequently, the blocking buffer was discarded, and the membrane was incubated with 1 mL of CAAT-CM overnight at 4°C. After several washing steps, the membrane was incubated with a biotinylated antibody cocktail overnight at 4°C. Following the incubation with the primary antibody and washing steps, horseradish peroxidase-conjugated streptavidin was added overnight at 4°C. Finally, signals were developed using chemiluminescence reagents, and the cytokine spot signal intensity was evaluated by subtraction from the background and normalization to positive controls on the same membrane using the ImageJ software (NIH Bethesda, Maryland, USA).

### Cell culture

The IBC cell line SUM-149, was a gift from Prof. Dr. Bonnie F. Sloane (Department of pharmacology, School of Medicine, Wayne State University, Detroit, 48201 MI, USA, and the non-IBC MDA-MB-231 cell line, was a gift from Prof. Dr. Martin Götte, a Münster University Hospital, Germany. SUM-149 cells were maintained in HAM’s**-**F12 medium containing 5% FBS, 1% penicillin/streptomycin, insulin, and hydrocortisone in a humidified atmosphere of 5% CO2 at 37°C. MDA-MB-231 cells were grown in a DMEM medium containing 10% FBS, 1% glutamine, and 1% penicillin/streptomycin in a humidified atmosphere of 5% CO2 at 37°C.

### Cell viability assay

Using 96-well plates, MDA-MB-231 (3.0 X 10^3^/well) and SUM-149 (5.0 X 10^3^/well) cells were incubated with complete DMEM and HAM’s-F12 medium for 48 h. Cells were starved in a culture medium without FBS for 24 h. Then, MDA-MB-231 and SUM-149 cells were treated with DMEM and HAM’s**-**F12 medium supplemented with 1% FBS in the absence (control-CM) or presence of 25%, 50%, and 75% of CAAT-CM for an additional 24 h. Subsequently, MTT (3-(4,5-dimethylthiazol-2-yl)-2,5-diphenyltetrazolium Bromide) was added, and the absorbance of treated cells compared with the control was measured at 570 nm using an Infinite®200 PRO NanoQuant instrument (Tecan, Zürich, Switzerland).

### Stimulation of the breast cancer cell lines MDA-MB-231 and SUM-149 with CAAT-CM

The cells were plated and at 80% confluence, were washed with warm PBS. They were then starved for 24 h in a serum-free growth medium. Subsequently, the cells were seeded in medium containing 1% FBS (Gibco, Waltham, Massachusetts, USA) and 25% CAAT-CM from patients with non-IBC and IBC for 24 h, respectively. In parallel, control cells were seeded in DMEM and HAM’s-F-12 medium containing 1% FBS. Finally, cells were lysed to isolate total RNA. All tissue culture medium were from Lonza (Basel, Switzerland), and supplies were from Greiner Bio-One (Frickenhausen, Germany), unless otherwise stated.

### RNA isolation and reverse transcription-quantitative polymerase chain reaction (RT-qPCR)

Total RNA was isolated from cultured cells (MDA-MB-231 and SUM-149) and fresh CAAT *ex-vivo* cultures using the TRIzol reagent (Ambion, Life Technologies) according to the manufacturer’s guidlines. About 1 μg RNA was reverse-transcribed into cDNA using the high capacity cDNA Reverse Transcription Kit (Applied Biosystems, Foster City, CA, USA) according to the manufacturer’s instructions after measurement of the concentration using Infinite®200 PRO NanoQuant (Tecan, Zürich, Switzerland). Next, using SYBR Green universal master mix Kit (Applied Biosystems, Foster City, CA, USA), *CD24*, *CD44*, E-cadherin, vimentin, *IL-6*, *Il-8*, and *MCP-1* gene expression levels were measured. The qPCR was performed in step-one plus amplification system (Applied Biosystems, San Francisco, CA, USA). Samples were incubated for initial denaturation at 95 °C for 10 min and then subjected to 40 PCR cycles as follows: 95°C for 15 sec, 55°-60°C for 1 min, followed by an amplification plot and melting curve to confirm specific product amplification. The ^ΔΔ^Ct method was used to quantify gene fold expression and data normalized to 18S rRNA for cell lines [41–43] and glyceraldehyde-3-phosphate dehydrogenase (GAPDH) for CAAT samples [44]. The selected reference genes are stable under our experimental conditions, as published before by the authors for both cell lines [45, 46].

### Wound healing assay

The MDA-MB-231 and SUM-149 cells were cultivated in a 6-well tissue culture plate until their growth rate reached a confluence of ~ 80%. After that, the cells were washed with PBS, and the monolayer was scratched gently with a 200 μL pipette tip across the center of the well. After creating the scratched area, wells were washed twice with PBS to remove the detached cells and incubated in 25% CAAT-CM for 24 h. Cell migration to the scratch area was monitored under the Zeiss Axiophot bright-field microscope at 0 and 24 h.

### Statistical analysis

All data are presented as the mean ± SEM except for the data in Additional file 1, which are presented as the mean ± SD as indicated. Data were analyzed using IBM SPSS version 15.0 for Windows (SPSS, Chicago, IL, USA). Differences among variables were evaluated using the chi-squared test and unpaired Student’s t-test. Pearson’s rank correlation test was used to analyze the correlations. The level of significance was set at *P* < 0.05.

## Results

### Establishment of a CAAT ex-vivo culture from patients with non-IBC and IBC

**Fig. 1:**
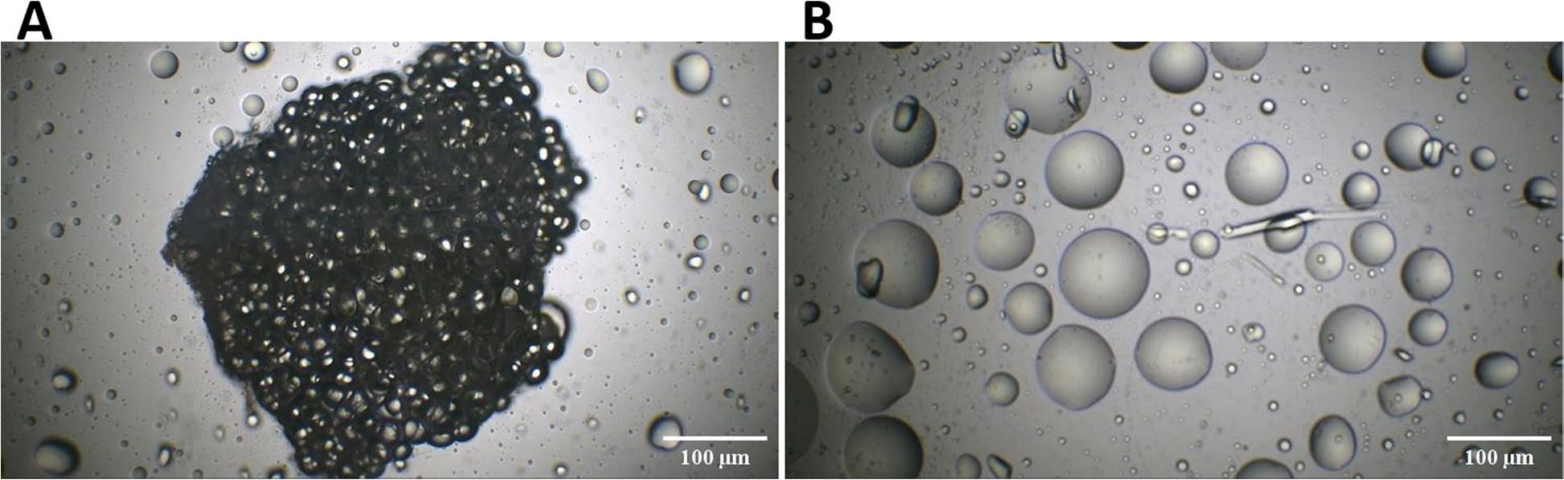
Photomicrographs representing *ex-vivo* culture CAAT immediately after surgery. **A** Representative microscopic images showing an ex-vivo culture of CAAT cultured in DMEM/F-12 medium supplemented with 10% FBS for 24 h in a humidified atmosphere of 5% CO2 at 37 °C. **B** Representative microscopic images focusing on the lipid droplets (LD_s_) secreted from CAAT after *ex-vivo* culture, scale bar 200 μm

CAAT that was cultured as an *ex-vivo* culture to enable cells to be organized into structures mimicking their *in-vivo* conditions (Fig. 1A) showed increased secretion of lipid droplets (LD_s_) (Fig. 1B). A lipid-specific dye (Oil red O) is used to stain lipid droplets, and the Images are provided Supplemental Figure 1.

### IL-6, IL-8, and MCP-1 are elevated in the secretome of IBC *vs.* non-IBC-derived CAAT

**Fig. 2:**
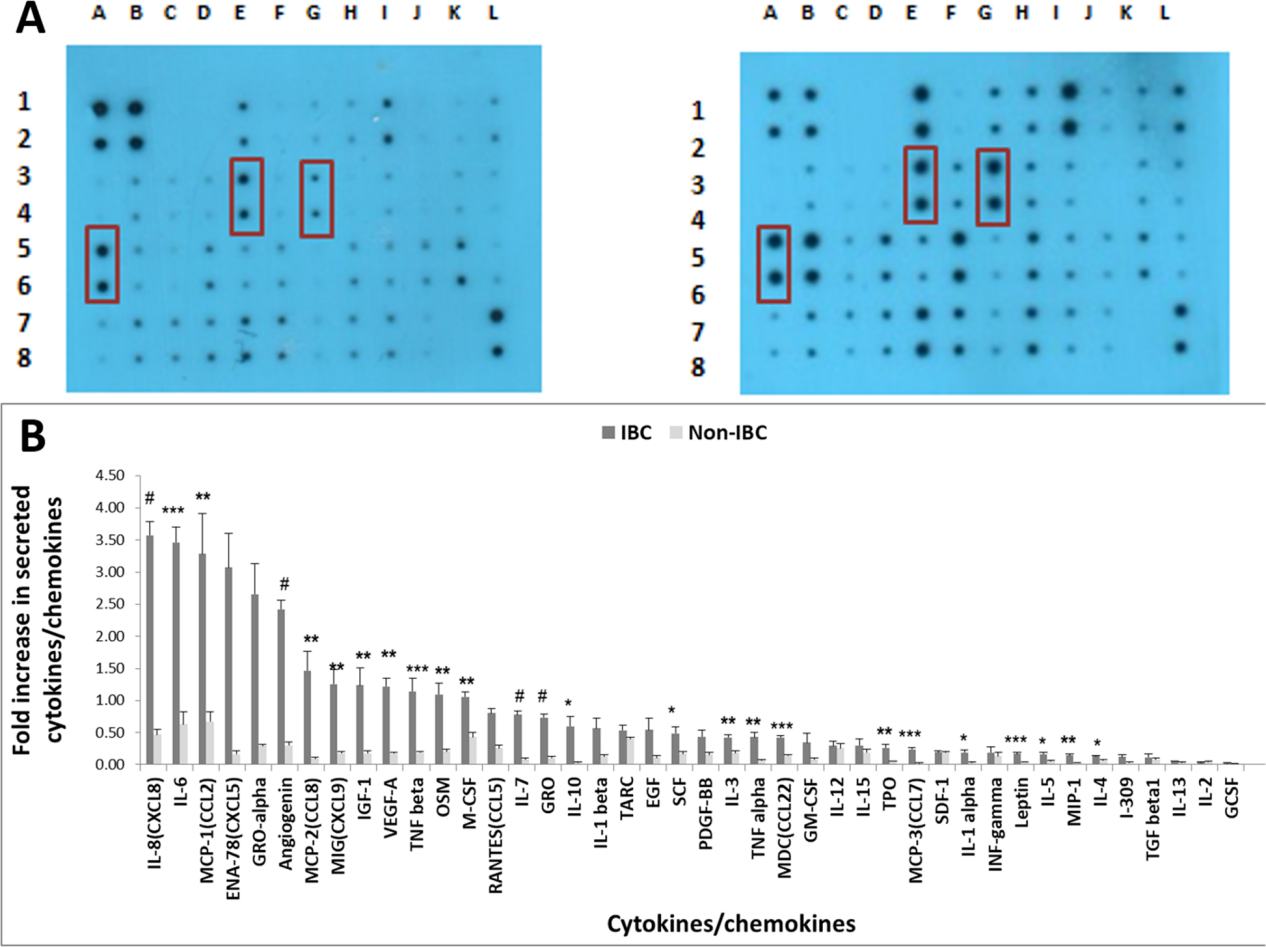
profiling of cytokines/chemokines/growth factors secreted from CAAT of non-IBC patients *vs.* IBC patients. **A** Representative RayBioTM cytokine antibody array 3 of secreted CAAT-CM from IBC compared to non-IBC patients after 24 h. Boxes indicate the localization of cytokines that show a statistically significant difference between non-IBC patients in the left panel and IBC patients in the right panel. **B** Bars represent the mean ± SD of the signal intensity value of each cytokine secreted by an *ex-vivo* culture of CAAT of non-IBC (n = 10) and IBC (n = 10). The cytokine array image was quantified using ImageJ software. The Student’s t-test was used to determine the significant differences in secretion levels of adipokines /growth factors between IBC versus non-IBC patients. ^*^Represents *P* < 0.05, ^**^represents *P* ≤ 0.01, ^***^represents *P* ≤ 0.001, and ^#^ represents *P* ≤ 0.0001 as determined by Student’s t-test

To identify the predominant cytokines and chemokines secreted by AT, the secretome of CAAT was profiled using the Ray-Bio^TM^ cytokine antibody array-C3 (RayBiotech, Inc. GA, USA). As depicted in Supplemental Table 1, there was a significant increase in 28 secreted cytokines in the secretome of CAAT from IBC compared with non-IBC samples**.** Interestingly, relative to the non-IBC group, *IL-6*, *IL-8,* and *MCP-1* were the predominant cytokines and chemokines in the secretome of CAAT from patients with IBC (*P* < 0.05 for all, Fig. 2).

### The IL-6, IL-8, and MCP-1 mRNAs are upregulated in ex-vivo culture of CAAT isolated from non-IBC vs. IBC samples.

**Fig. 3:**
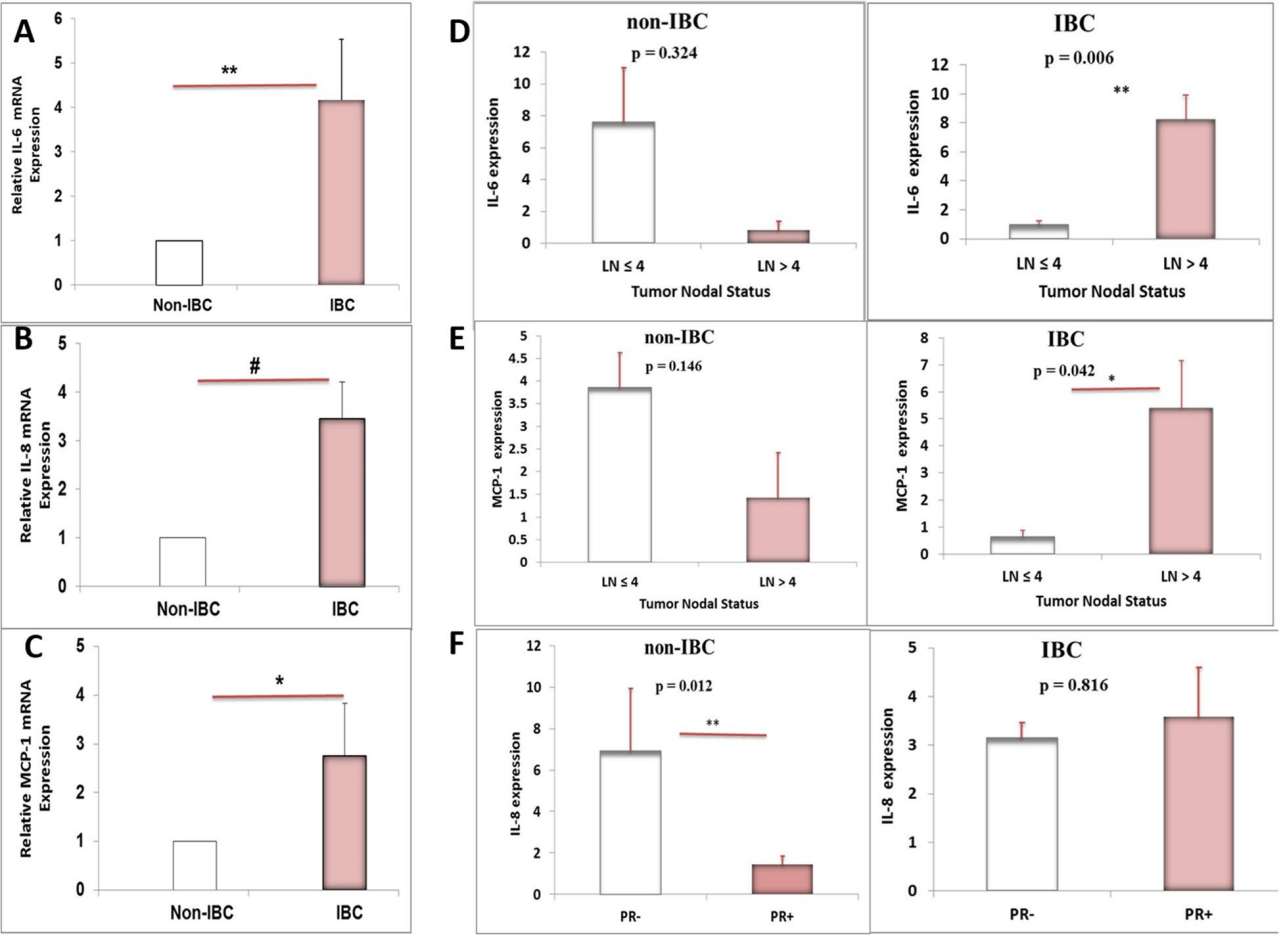
qRT-PCR comparing the expression level of the predominant cytokines secreted by CAAT isolated from non-IBC versus IBC patients. **A** A significantly higher mRNA expression level of *IL-6*, (**B**) *IL-8*, and (**C**) *MCP-1* of CAAT *ex-vivo* culture from patients with IBC *vs.* non-IBC by 4.17, 3.46, and 2.75 folds, respectively. ^**^ *P* < 0.01, ^#^ *P* < 0.0001.^*^ *P* < 0.05 as determined by unpaired Student t-test. Quantification of target genes mRNA was expressed relative to the housekeeping gene (GAPDH) mRNA. All bar graphs represent fold change+/−SEM (n of non-IBC = 21 and n of IBC = 11). **D** A significant up-regulation in *IL-6* mRNA expression of obese IBC patients that had lymph node (LN) metastasis (*n* = 10, *P* = 0.006) in the right panel and no change in *IL-6* mRNA expression of obese non-IBC patients that have LN metastasis (*n* = 17, *P* = 0.324) in the left panel was observed. **E** The transcript level of MCP-1 is significantly up-regulated in obese IBC patients that have LN metastasis (*n* = 10, *P* = 0.042) in the right panel, but not in obese non-IBC patients that have LN metastasis (*n* = 17, *P* = 0.146) in the left panel. **F** A significant down-regulation in *IL-8* mRNA expression of obese non-IBC patients that express PR receptor (*n* = 21, *P* = 0.012) in the left panel but not in obese IBC patients that express PR receptor (*n* = 10, *P* = 0.816) in the right panel

Using qRT-PCR, the expression of *IL-6*, *IL-8*, and *MCP-1* was further verified at the transcript level in explants of CAAT from both the non-IBC and IBC groups. The qRT-PCR analysis showed a significant upregulation of *IL-6* (4.17-fold, *P* = 0.004; Fig. 3A), *IL-8* (3.46-fold, *P* = 0.0001; Fig. 3B), and *MCP-1* (2.75-fold, *P* = 0.039; Fig. 3C) mRNAs in IBC *vs.* non-IBC samples.

Moreover, significant upregulation of the in *IL-6* mRNA was detected (*P* = 0.006) in IBC patients with obesity who had lymph node (LN) metastasis (*n* = 10; Fig. 3D, the left panel) when compared with the non-IBC group (*n* = 17) for which its expression was not changed (*P* = 0.324; Fig. 3D, right panel). Similarly, significant upregulation of the *MCP-1* mRNA (*n* = 10, *P* = 0.042) was observed in patients with obesity and IBC who had LN metastasis (Fig. 3E, left panel). Conversely, there was no difference in *MCP-1* mRNA expression in patients with obesity and non-IBC who have LN metastasis (n = 17, *P* = 0.146; Fig. 3E, right panel). Furthermore, significant (*P* = 0.012) downregulation of the *IL-8* mRNA was detected in patients with obesity and non-IBC who expressed the progesterone (PR) receptor (*n* = 21; Fig. 3F, left panel). However, there was no change in *IL-8* mRNA in patients with obesity and IBC who expressed the PR receptor (*n* = 10, *P* = 0.816; Fig. 3F, right panel).

### CAAT-CM effect on the viability of the MDA-231 and SUM-149 cell line

**Fig. 4:**
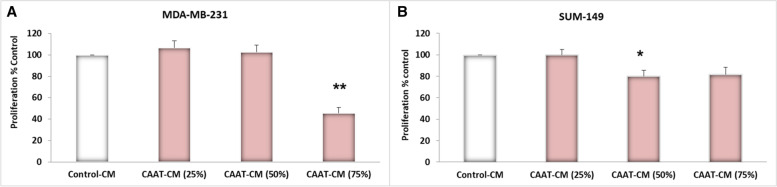
CAAT-CM effect on MDA-231 and SUM-149 cells proliferation**.** MDA-MB-231 and SUM-149 cells were exposed to different concentrations (25%, 50%, and 75%) of CAAT-CM from obese non-IBC and IBC patients for 24 h *vs.* control cells. (**A**) No effect was observed in MDA-MB-231 cells growth when seeded in 25%, 50% CAAT-CM, and 75% CAAT-CM downregulated cell proliferation significantly. (**B**) The proliferation of SUM-149 cells was downregulated significantly by the presence of 50% CAAT-CM, but it was not affected by 25% or 50% CAAT-CM. MTT was used to assess the proliferation of cells. Bar graphs represent the old change ± SEM. ^**^Represents *P* ≤ 0.01 as determined by the Student’s t-test

To determine the effect of medium conditioned by an *ex-vivo* culture of CAAT from patients with obesity in the non-IBC and IBC groups on the proliferation of MDA-231 and SUM-149 cells, respectively, cells were seeded in the absence (control-CM) or presence of 25%, 50%, and 75% CAAT-CM diluted with DMEM-F12 medium for 24 h. There was no change in the proliferation of MDA-231 and SUM-149 cells treated with 25% CAAT-CM compared with the control-CM (*P* = 0.555 and *P* = 0.98), respectively. Additionally, a significant decrease in the proliferation of SUM-149 cells was observed after incubation with 50% CAAT-CM (*P* = 0.039), whereas proliferation was not changed in MDA-231 cells treated with 50% CAAT-CM compared with control-CM. A significant decrease in the proliferation of MDA-231 cells was detected in the presence of 75% CAAT-CM (*P* = 0.003), whereas the proliferation of SUM-149 cells was not affected by the presence of 75% CAAT-CM as shown in Fig. 4.

### CAAT-CM of the non-IBC and IBC groups altered the expression of CSC and EMT markers in MDA-MB-231 and SUM-149 cells

**Fig. 5:**
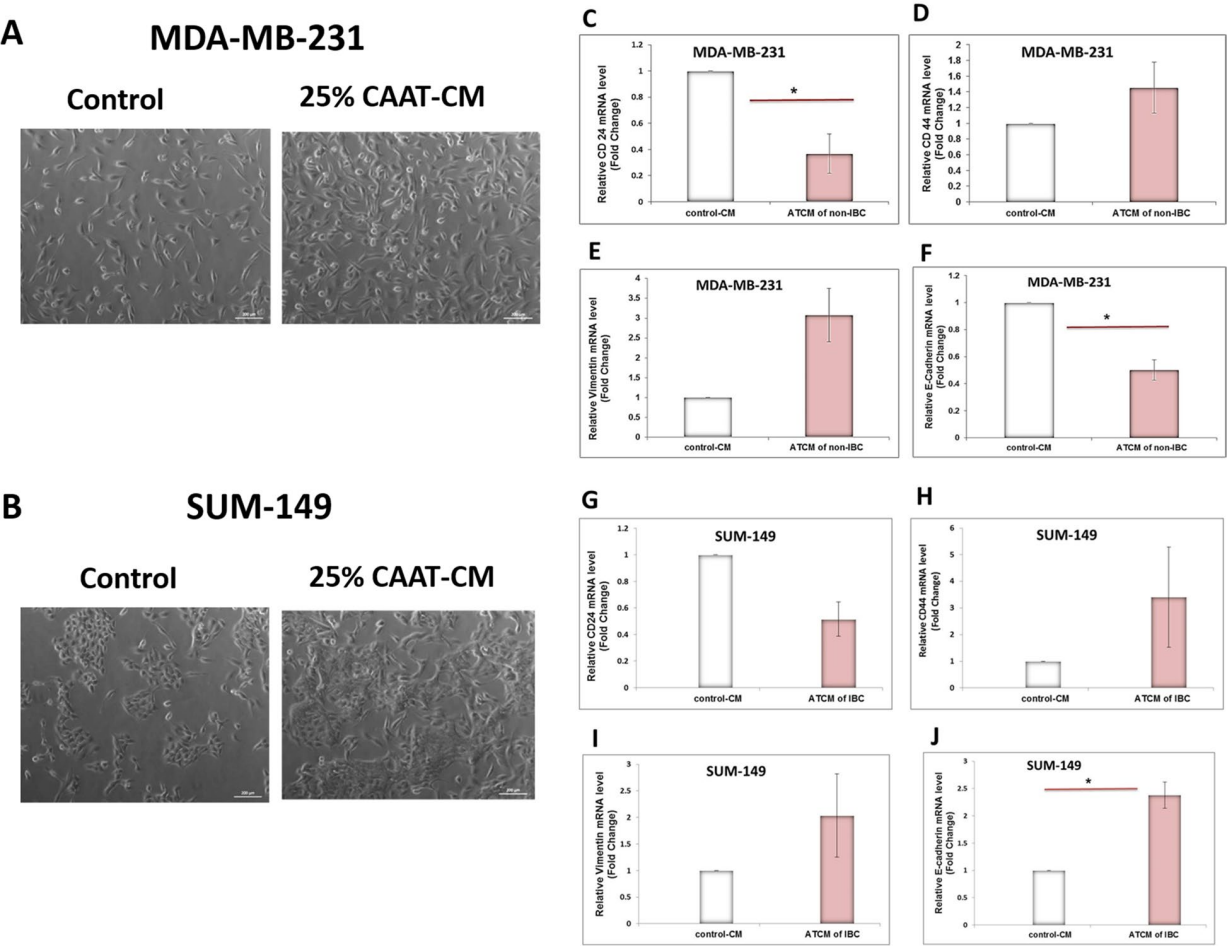
CAAT-CM effect on BC cell morphological features, CSC, and EMT markers gene expression**.** Photomicrographs represent the morphological change of (**A**) MDA-MB-231 and (**B**) SUM-149 BC cells after treatment with 25 % CAAT-CM for 24 h. MDA-MB-231 cells showed elongated spindle-shaped morphology compared to untreated cells. In contrast, SUM-149 cell morphology clustered as aggregates. Cell morphology was observed by phase-contrast microscopy. Scale bar, 200 μm. (**C**) The mRNA expression of *CD24* was significantly downregulated in the treated MDA231 with CAAT-CM compared with the control. **D** no change in the mRNA expression of *CD44* was observed, **E** the mRNA expression of vimentin tended to be significantly upregulated in the treated MDA231 cells with CAAT-CM compared with the control by (3.08 fold), and **F** the mRNA expression of E-Cadherin was significantly downregulated in the treated MDA231 cells with CAAT-CM compared with the control. **G** The mRNA expression of *CD24* tended to be significantly downregulated in the treated SUM149 cells, **H** No change in the mRNA expression of CD44 and **I** vimentin was observed, and **J** the mRNA expression of E-Cadherin was significantly upregulated in the treated SUM149 cells with CAAT-CM compared with the control by (2.38 fold). ^*^P < 0.05 as determined by Student’s t-test. MDA-231 and SUM-149 cells were seeded in the absence (control) and presence of 25% CAAT-CM for 24 h (treated). Relative quantification of mRNA expression was analyzed using qRT-PCR (n of non-IBC = 18 and n of IBC = 9). Quantification of target genes mRNA was expressed relative to the housekeeping gene (18S) mRNA. The data shown are the mean ± SEM

Notably, cancer-associated adipocytes in the breast contribute to cancer growth by communicating with cancer cells by releasing adipokines [47]. MDA-MB-231 cells treated with non-IBC-CAAT-CM exhibited an elongated spindle-shaped morphology compared with untreated cells, whereas SUM-149 cells treated with IBC-CAAT-CM clustered into aggregates as observed in Fig. 5A and B, respectively.

The effect of the secretome of CAAT isolated from patients with obesity and BC was investigated on the mRNA expression levels of CSC markers, such as *CD24* and *CD44* as well as EMT markers, such as vimentin and E-cadherin, was investigated in MDA-231 and SUM-149 cells using qRT-PCR. The results showed that the CAAT-CM significantly downregulated the mRNAs of the surface marker *CD24* (0.37-fold, *P* = 0.024; Fig. 5C) and epithelial E-cadherin marker (0.50-fold, *P* = 0.003; Fig. 5F) of MDA-231 cells upon stimulation with CAAT-CM from non-IBC groups. However, no change was observed in surface marker *CD44* (1.45-fold, *P* = 0.371; Fig. 5D). Moreover, the vimentin mesenchymal marker tended to be significantly upregulated in MDA-231 upon stimulation (3.08-fold, *P* = 0.074; Fig. 5E). Moreover, the expression level of the mRNA for the surface *CD24* marker tended to be significantly downregulated (0.76-fold, *P* = 0.098; Fig. 5G) upregulated in SUM-149 upon stimulation with CAAT-CM from IBC groups. In contrast, no changes were observed in the level of expression of the mRNAs for the *CD44* (3.40-fold, *P* = 0.478; Fig. 5H) and vimentin (2.04-fold, *P* = 0.463; Fig. 5I) markers. Besides, the results showed that the epithelial E-cadherin marker was significantly upregulated (2.38-fold, *P* = 0.036; Fig. 5J).

### CAAT-CM effect on the motility of the MDA-231 and SUM-149 cell lines

**Fig. 6:**
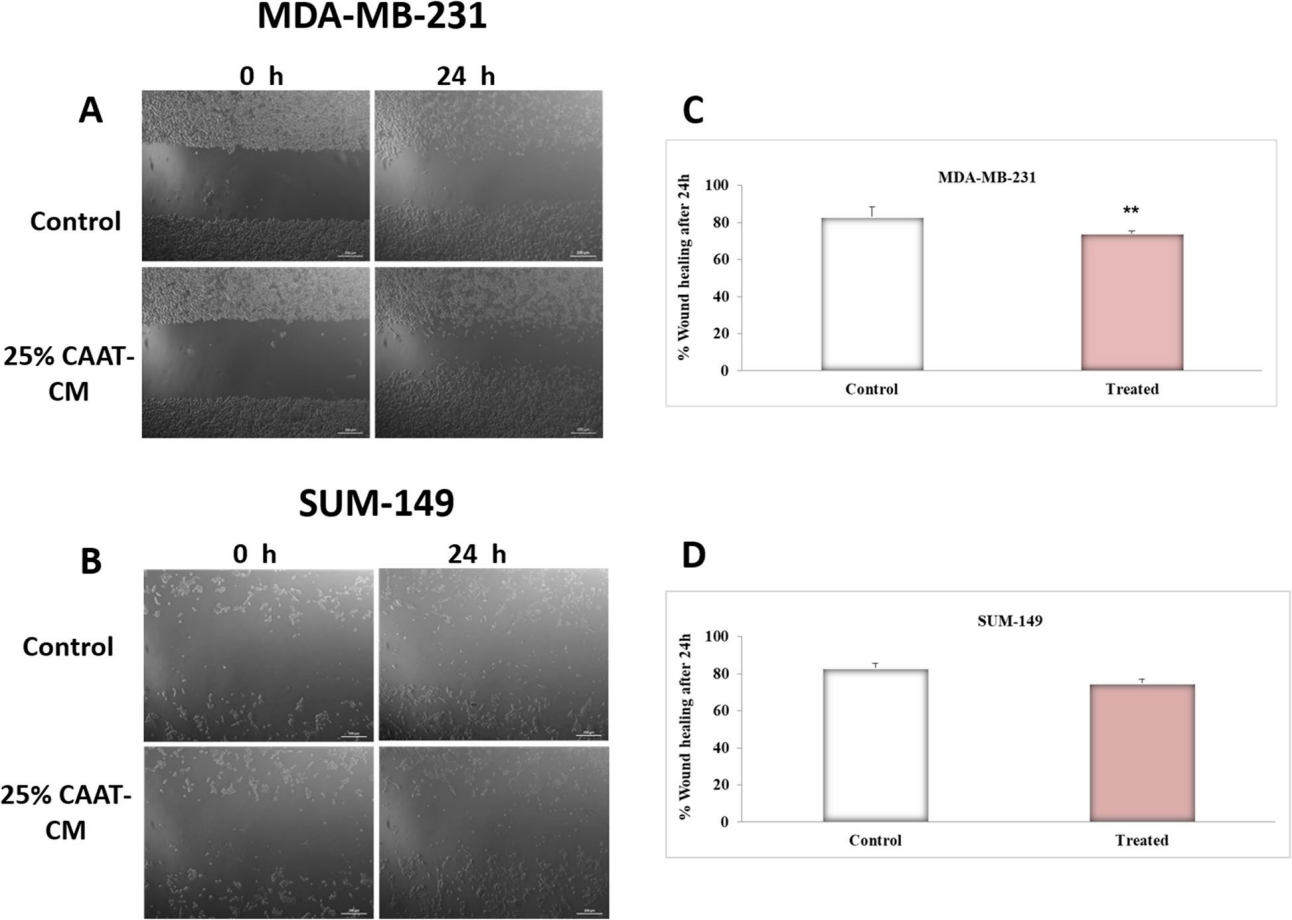
CAAT-CM effect on MDA-231 and SUM-149 cells motility. Representative photographs of (**A**) MDA-MB-231 and (**B**) SUM-149 BC cells in the wound healing assay at 0 hr and at 24 h. Scale bar, 200 μm. (**C**) The percentage of wound closure assay reveals a higher migratory potential of MDA-MB-231 cells compared with the control upon stimulation with 25% CAAT-CM. (**D**) The percentage of wound closure assay tended to show significance in SUM-149 cell migration compared with the control upon stimulation with 25% CAAT-CM. The area of wound closure was imaged at the indicated times and measured using the ImageJ software. Bar graphs represent a fold change ± SEM. ^**^Represents *P* ≤ 0.01 as determined by the Student’s t-test

MDA-MB-231 cells showed a significant acceleration of scratch wound closure by the presence of 25% CAAT-CM compared with control (*P* = 0.004) as shown in Fig. 6C. Besides, SUM-149 cells showed a trend significance acceleration of scratch wound closure in response to 25% CAAT-CM compared with control (*P* = 0.087) as clear in Fig. 6D.

## Discussion

CAAT is an influential player in BC progression, as this type of cancer grows in the AT vicinity [27]. As a consequence, numerous studies have begun to provide insights into the role of AT in BC patients with obesity and its impact on BC migration and development [27, 35, 48, 49].

In fact, dysfunctional AT is associated with an increased risk for various cancers, including the esophageal adenocarcinoma, pancreas, colon, rectum, prostate, and ovarian cancer, as well as BC, in patients with obesity via the expression of a variety of pro-inflammatory adipocytokines and other factors that play a pivotal role in cancer metastasis [50]. Among these adipokines, *IL-6*, *IL-8*, and *MCP-1* are released by adipocytes [50, 51]. In the present study, adipose tissues were isolated from patients with non-IBC and IBC and cultured *ex-vivo*; this experiment showed that adipocytokines are a potential candidate linking obesity with BC progression.

The present study aimed to profile the secretome of CAAT obtained during MRM as an *ex-vivo* patient tissue culture. The *ex-vivo* patient tissue culture provides mammary epithelial cells with a basement membrane-like gel, which enables cells to be organized into structures that mimick the *in-vivo* conditions and maintain their growth [52]. Moreover, cell–matrix interactions that modulate the cytoskeleton are enabled when cells grow as an *ex-vivo* culture [53]. Lastly, a growing body of evidence has suggested that the *ex-vivo* culture 3D model can preserve cell physiological functions to a greater extent than do those grown in 2D culture [54].

The aims of the present study also included the measurement of the major cytokines and chemokines in the culture medium of CAAT collected from obese non-IBC patients *vs.* obese IBC patients. The cytokine array showed a significantly higher level of a panel of 28 cytokines secreted by CAAT of obese IBC patients *vs.* non-IBC patients, with a more than three-fold change observed for the predominant cytokines *IL-6*, *IL-8*, and *MCP-1.* Furthermore, the levels of expression of the *IL-6*, *IL-8*, and *MCP-1* mRNAs were upregulated in IBC- and non-IBC-derived CAAT. To our knowledge, the present study demonstrated for the first time that the CAAT of obese IBC groups had significantly higher levels of the *IL-6*, *IL-8*, and *MCP-1* mRNAs and protein levels in CAAT culture medium compared with that of non-IBC obese groups. This result agrees with previous studies that demonstrated that the upregulation of *IL-6*, *IL-8*, and *MCP-1* is correlated with a poor prognosis of BC in patients with obesity [55]. Regarding the patients, they had a poorer compared with those the non-IBC [9].

It is widely acknowledged that adipocytes play an influential role in the promotion of cancer growth and progression [56]. Additionally, it has been shown that the release of the *IL-6* and *IL-8* cytokines in metabolic disorders, such as obesity, is increased to mediate the crosstalk between adipocytes and BC cells within the tumor microenvironment [37]. Adipocytes become fertile soil for tumors in patients with, thus allowing them to spread distantly for distant and protecting them from therapeutic treatment [56].

IL-6 has been identified as a marker linked to increased lymph node in patients with BC [57]. Interestingly, a higher expression of IL-6 positively correlates with lymph node in obese IBC groups but not in non-IBC groups enrolled in the present study. These findings are in accordance with the study that highlighted that the IL-6 level in CAAT increases with increasing lymph node involvement [50]. In this regard, IL6 may contribute to the increased IBC aggressiveness and progression via stimulating aromatase expression in AT, thus stimulating estrogen synthesis [58].

Besides, the results analyses provide a significant positive correlation between the lymph node metastasis and *MCP-1* transcription level in the obese IBC patients but not in non-IBC patients. This is in accordance with the study mentioned *MCP-1* is associated with lymph node metastasis and tumor malignancy of the breast [59].

To extend these findings to *in-vitro* cultures and to understand better the effect of the secretome of CAAT on the expression of CSC markers such as *CD24* and *CD44* as well as EMT markers such as vimentin and E-cadherin, in both non-IBC (MDA-231) and IBC (SUM-149) cell lines, first, the best concentration of CAAT-CM was determined and shown not to be toxic to the cells using an MTT assay. The data revealed no changes in MDA-MB-231 cell growth was observed when seeded in 25% and 50% CAAT-CM, whereas 75% CAAT-CM was toxic to the BC cell line and decreased its viability. Additionally, there was no effect on SUM-149 cell proliferation in the presence of 25% and 75% CAAT-CM, whereas 50% CAAT-CM decreased cell proliferation. The present results disagree with other studies showing an absence of changes in the proliferation of MCF-10A, HBL100, MCF-7, and IBH-7 cells when incubated with 50 % and 75% ATCM compared with the control [2]. The differences between the present study and the previous study may be attributed to the different types of cell lines used here or the biological properties of Egyptian patients with BC compared with other patients populations. In this regard, we suggest that 25% CAAT-CM is the best concentration based on the data evaluation and that it is preferable to use the lowest amount of CAAT-CM to preserve the CAAT-CM of patients with BC for further experiments.

The data revealed that CAAT-CM significantly downregulated the mRNA for the *CD24* surface marker in MDA-231 cells and tended to significantly downregulates it in SUM-149 cells. A pioneering study has indicated that CD24 is a significant marker linked to tumor metastasis, increased cell proliferation, motility, and invasiveness of abnormal fat accumulation in the body, and is associated with poor clinical outcomes in breast carcinomas [60].

Moreover, vimentin, which is a mesenchymal marker, tended to be significantly upregulated in MDA-231 cells upon stimulation, whereas no change was observed in SUM-149 cells. Such findings are consistent with other reports demonstrating that the co-culture of MDA-231 cells with tumor cells induces the EMT phenotype, which is characterized by the upregulation of the vimentin mesenchymal marker [61, 62]. Furthermore, the data agree with another study that indicated that vimentin expression is necessary for the migration and invasion of BC and that its blockage, in turn, triggers a decrease in BC cell migration and invasiveness [63]. These activities were mediated by PCKα/PP2A/C-SRC and JAK-STAT signaling pathway activation, resulting in STAT3 phosphorylation [64].

Furthermore, the present results revealed that the secretome of CAAT upregulated the E-cadherin epithelial marker in SUM-149 cells and downregulated it in MDA231 cells, which is the hallmark of tumor emboli formation in patients with IBC. This data agreed with another study carried out by our lab, which showed that E-cadherin was overexpressed in the IBC compared with the non-IBC group [13]. Additionally, such findings are consistent with the opposite role played by E-cadherin in patients with non-IBC and IBC. The increased tumor proliferation and metastasis observed in patients with non-IBC are attributed to the loss of E-cadherin expression [65], whereas the disease aggressiveness detected in patients with IBC is attributed to upregulation of the E-cadherin epithelial marker [66]. This may be ascribed to the role of the E-cadherin molecule in allowing carcinoma cells to migrate as a clump of cells that block blood vessels in different organs, causing organ failure and death, as mentioned in a previous study [67]. In this regard, the data indicate that CAAT-CM may play a role in the stem cell properties of cancer cells, thus potentially triggering cancer recurrence.

### Study Strengths and Limitations

This study had the following strengths: (1) it was the first to culture CAATs obtained during MRM or curative surgery as an *ex-vivo* patient tissue culture, and (2) it allowed identification of the major cytokines in CAAT culture medium of obese IBC patients compared with those with non-IBC.

However, this study also had several limitations: (1) this intriguing observation requires further study to determine whether the prominent IL-6, IL-8, and MCP-1 adipokines affect CSCs by knockdown them, and future studies using larger patient cohorts will help define their total prognostic and predictive value in IBC, and (2) the interpretation of the results obtained for the *IL-6*, *IL-8*, and *MCP-1* cytokines in correlation with BMI was limited because the patients were nearly obese due to the increased obesity rate in Egypt [68], which may contribute to BC. Therefore, only obese patients with BC were enrolled in the present study exclusively.

## Conclusions

The study findings suggest that secretions from CAAT collected from the tumor margin of patients with obesity increased IBC aggressiveness by regulating the CSC and EMT phenotypes and are considered an important prognostic factor for BC recurrence. Hence, targeting IL-6, IL-8, and MCP-1 may represent a therapeutic option for obese IBC patients.

The study validates the usage of CAAT as an ex-vivo model that mimics in vivo patients’ tumor microenvironment to assess the key inflammatory meditators and study their effect on modulating gene expression of stem cell markers associated with cancer poor prognosis and relapse.

Herein, CAAT of tumor margins of obese IBC patients is characterized by the secretion of inflammatory mediator IL-6, IL-8, and MCP-1 that plays a significant role in proliferation, motility, invasion, induction of proteolytic activity, and drug resistance in the tumor microenvironment as described before by the authors (Mohamed et al., 2014 and 2021).

The *ex-vivo* model of CAAT can be used in preclinical studies for screening CAAT derived mediators (adipokines) in different types of cancer, not only breast cancer, and identify how CAAT plays a crucial in cancer relapse and metastasis in obese patients

## Supplementary Information

**Additional file 1:** Supplemental Figure 1. Photomicrographs representing ex-vivo cultured CAAT and secreted lipid droplets stained with oil red O.

**Additional file 2:** Supplemental Table S1. A statistical difference in CAAT secretome levels of IBC vs. non-IBC patients.

## Data Availability

The data related to this study can be available from the corresponding author upon reasonable request.

## Abbreviations

IBC: Inflammatory breast cancer
BC: Breast cancer
CAAT: Cancer-associated adipose tissue
AT: Adipose tissue
CSC_S_: cancer stem cells
EMT: Epithelial-mesenchymal transition
BMI: Body Mass Index
ASCs: Adipose stromal/stem cells
TME: Tumours Micronvironmental
CM_S_: Conditioned medium
*IL-6*: Interleukin (IL)-6
IL-8: Interleukin (IL)-8
*MCP-1/ CCL2*: monocyte chemoattractant protein 1
GAPDH: glyceraldehyde-3-phosphate dehydrogenase
LD: lipid droplet
MTT: 3-(4,5-Dimethylthiazol-2-yl)-2,5-diphenyltetrazolium bromide
Vim: vimentin
MRM: modified radical mastectomy
PBS: phosphate-buffered saline
