# Supplementary material for: Role of adipose tissue-derived cytokines in the progression of inflammatory breast cancer in patients with obesity
Source: Lipids Health Dis. 2022 Aug 4;21:67. doi: 10.1186/s12944-022-01678-y (PMC9351154; doi:10.1186/s12944-022-01678-y)
Supplement: Supplementary file 1 — Additional file 1: Supplemental Figure 1. Photomicrographs representing ex-vivo cultured CAAT and secreted lipid droplets stained with oil red O. [file 12944_2022_1678_MOESM1_ESM.docx]

**Supplemental Figure 1** of step to validation of that these are indeed lipid droplets using Oil red O.


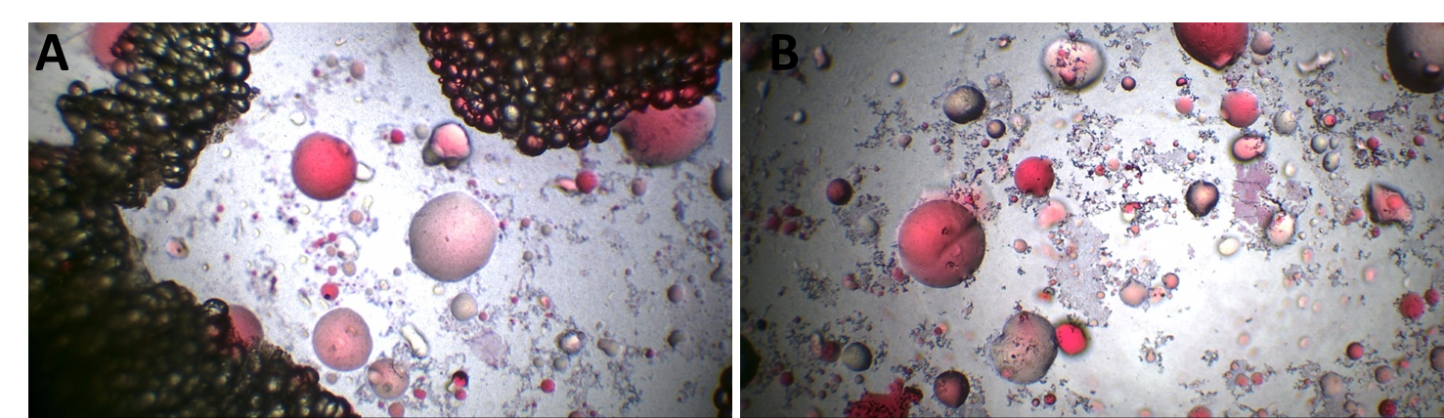


## Supplementary Figure 1. Photomicrographs representing *ex-vivo* cultured CAAT and secreted lipid droplets stained with oil red O.

(A) Representative photographs showing an oil red O staining of ex-vivo cultured CAAT and the surrounding secreted lipid droplets (LD_s_). (B) Representative photographs focusing on the stained lipid droplets (LD_s_) secreted from CAAT *ex-vivo* culture with oil red O. The images were visualized by light microscopy.
