# Supplementary material for: Role of adipose tissue-derived cytokines in the progression of inflammatory breast cancer in patients with obesity
Source: Lipids Health Dis. 2022 Aug 4;21:67. doi: 10.1186/s12944-022-01678-y (PMC9351154; doi:10.1186/s12944-022-01678-y)
Supplement: Supplementary file 2 — Additional file 2: Supplemental Table S1. A statistical difference in CAAT secretome levels of IBC vs. non-IBC patients. [file 12944_2022_1678_MOESM2_ESM.docx]

Supplemental Table S1. A statistical difference in CAAT secretome levels of IBC vs. non-IBC patients

| Cytokines symbol | Cytokines Name | Non-IBC  (N = 10) | IBC  (N = 10) | | | *P* value |
| --- | --- | --- | --- | --- | --- | --- |
| IL-2 | Interleukin-2 | 0.04 ± 0.03 | | 0.04 ± 0.01 | 0.60 | |
| MCP-1(CCL2) | Monocyte chemoattractant protein-1 | 0.67 ± 0.31 | | 3.28 ± 1.26 | 0.01^**^ | |
| TNF alpha | Tumour Necrosis Factor alpha | 0.07 ± 0.03 | | 0.43 ± 0.15 | 0.01^**^ | |
| IL-3 | Interleukin-3 | 0.18 ± 0.08 | | 0.43 ± 0.07 | 0.01^**^ | |
| MCP-2(CCL8) | Monocyte chemoattractant protein-1 | 0.09 ± 0.04 | | 1.47 ± 0.61 | 0.01^**^ | |
| TNF beta | Tumour Necrosis Factor beta | 0.19 ± 0.04 | | 1.14 ± 0.41 | 0.01^**^ | |
| IL-4 | Interleukin-4 | 0.07 ± 0.03 | | 0.13 ± 0.03 | 0.03^*^ | |
| MCP-3(CCL7) | Monocyte chemoattractant protein-3 | 0.02 ± 0.01 | | 0.24 ± 0.06 | 0.001^***^ | |
| EGF | Epidermal growth factor | 0.11 ± 0.06 | | 0.54 ± 0.36 | 0.08 | |
| IL-5 | Interleukin-5 | 0.06 ± 0.02 | | 0.16 ± 0.06 | 0.04^*^ | |
| M-CSF | macrophage colony-stimulating factor | 0.43 ± 0.15 | | 1.06 ± 0.15 | 0.002^**^ | |
| IGF-1 | Insulin-like growth factor-1 | 0.18 ± 0.07 | | 1.24 ± 0.54 | 0.01^**^ | |
| ENA-78(CXCL5) | epithelial-derived neutrophil-activating peptide 78 | 0.17 ± 0.09 | | 3.07 ± 1.05 | 0.0031^**^ | |
| IL-6 | Interleukin-6 | 0.63 ± 0.38 | | 3.47 ± 0.47 | 0.0002^***^ | |
| MDC(CCL22) | Macrophage-Derived Chemokine | 0.14 ± 0.03 | | 0.42 ± 0.06 | 0.0004^***^ | |
| Ang | Angiogenin | 0.30 ± 0.12 | | 2.42 ± 0.28 | 0.0000^#^ | |
| GCSF | Granulocyte-colony stimulating factor | 0.02 ± 0.00 | | 0.02 ± 0.02 | 0.97 | |
| IL-7 | Interleukin-7 | 0.08 ± 0.04 | | 0.78 ± 0.10 | 0.0000^#^ | |
| MIG(CXCL9) | Monokine induced by gamma | 0.18 ± 0.06 | | 1.25 ± 0.46 | 0.01^**^ | |
| OSM | Oncostatin M | 0.20 ± 0.08 | | 1.09 ± 0.35 | 0.01^**^ | |
| GM-CSF | Granulocyte-macrophage colon stimulating factor | 0.08 ± 0.04 | | 0.34 ± 0.29 | 0.17 | |
| IL-8(CXCL8) | Interleukin-8 | 0.47 ± 0.16 | | 3.57 ± 0.42 | 0.0000^#^ | |
| MIP-1 | Macrophage Inflammatory Proteins | 0.03 ± 0.01 | | 0.15 ± 0.05 | 0.01^**^ | |
| TPO | Thyroid Peroxidase precursor | 0.05 ± 0.01 | | 0.26 ± 0.10 | 0.01^**^ | |
| GRO | Growth-regulated protein | 0.10 ± 0.05 | | 0.73 ± 0.11 | 0.0001^#^ | |
| IL-10 | Interleukin-10 | 0.03 ± 0.02 | | 0.60 ± 0.30 | 0.02^*^ | |
| (CCL5) | RANTES | 0.26 ± 0.08 | | 0.80 ± 0.15 | 0.0013^***^ | |
| VEGF-A | Vascular endothelial growth factor A | 0.18 ± 0.02 | | 1.22 ± 0.26 | 0.0005^***^ | |
| GRO-alpha | Growth-regulated alpha | 0.29 ± 0.05 | | 2.66 ± 0.95 | 0.0050^**^ | |
| IL-12 | Interleukin-12 | 0.25 ± 0.14 | | 0.29 ± 0.16 | 0.77 | |
| SCF | F-box containing complex | 0.17 ± 0.05 | | 0.49 ± 0.21 | 0.04^*^ | |
| PDGF-BB | Platelet-derived growth factor-BB | 0.16 ± 0.05 | | 0.44 ± 0.21 | 0.06 | |
| I-309 | chemokine (C-C motif) ligand 1 | 0.03 ± 0.02 | | 0.12 ± 0.07 | 0.06 | |
| IL-13 | Interleukin-13 | 0.03 ± 0.03 | | 0.05 ± 0.01 | 0.34 | |
| SDF-1 | stromal cell-derived factor 1 | 0.19 ± 0.03 | | 0.19 ± 0.04 | 1.00 | |
| Lep | Leptin | 0.04 ± 0.01 | | 0.17 ± 0.05 | 0.003^**^ | |
| IL-1 alpha | Interleukin 1 alpha | 0.03 ± 0.01 | | 0.19 ± 0.09 | 0.03^*^ | |
| IL-15 | Interleukin-15 | 0.19 ± 0.10 | | 0.30 ± 0.21 | 0.43 | |
| TARC | Thymus and activation-regulated chemokine | 0.40 ± 0.05 | | 0.54 ± 0.16 | 0.21 | |
| IL-1 beta | Interleukin 1 beta | 0.13 ± 0.03 | | 0.57 ± 0.31 | 0.05 | |
| INF-gamma | Interferon Gamma | 0.14 ± 0.11 | | 0.19 ± 0.19 | 0.69 | |
| TGF beta1 | Transforming growth factor beta 1 | 0.09 ± 0.03 | | 0.12 ± 0.10 | 0.62 | |
| Data are expressed as mean ± SD  ^*^Represents P < 0.05, ^**^represents P ≤ 0.01, ^***^represents P ≤ 0.001, and ^#^represents p ≤ 0.0001 as determined by Student’s t-test. | | | | | | |
